# Surgical management of acute distal biceps tendon rupture associated with contralateral radial palsy

**DOI:** 10.11604/pamj.2015.22.258.7408

**Published:** 2015-11-18

**Authors:** Badr Ennaciri, Emmanuel Beaudouin, Mustapha Mahfoud, Mohamed Saleh Berrada, Eric Montbarbon

**Affiliations:** 1 Department of Orthopedics, Avicenna University Hospital, Rabat, Morocco; 2 Department of Orthopedics, Chambéry Hospital, Chambéry, France

**Keywords:** Distal biceps tendon, trans-osseous anchoring, supination strength

## Abstract

Acute distal biceps tendon rupture constitute a rare lesion of biceps injuries, typically, easy to diagnosis after lifting a heavy object. Treatment is controversial, nonoperative for sedentary and elderly patients; surgical for young and active individuals. Many operative techniques are described, they all aim to restore an excellent strength of flexion and supination. We opted for one-incision method and fixation using trans-osseous anchoring for our patient, because we are convinced that is a simpler and safer technique. Postoperative rehabilitation, after a period of elbow immobilization, must be operated for returning to full activity. Biceps tendon repair has permitted to our patient who suffer from right upper limb handicap due to radial nerve palsy, recuperating the lost strength and force in his dominant limb and maintaining some quality of life.

## Introduction

Acute distal biceps tendon rupture is rare than proximal lesions, it represents 3% of biceps injuries according to Gilcreest and Albi [1]. Biomechanically important, it contributes on elbow flexion and forearm supination. The diagnosis is clinically easy in complete rupture, and confirmed by MRI. Many therapeutic options can be proposed (from therapeutic abstention to surgical repair) and various approach are used to repair this lesion. Divergent results are observed after surgery, in term of elbow and forearm motions. We report the case of a patient who sustained acute complete distal tendon rupture in his left arm associated with sequelae of radial nerve palsy in contralateral upper limb. He was managed surgically by single anterior approach and trans-osseous anchoring of distal tendon in bicipital tuberosity. The aim of surgery repair was both esthetic and functional.

## Patient and observation

**Figure 1:**
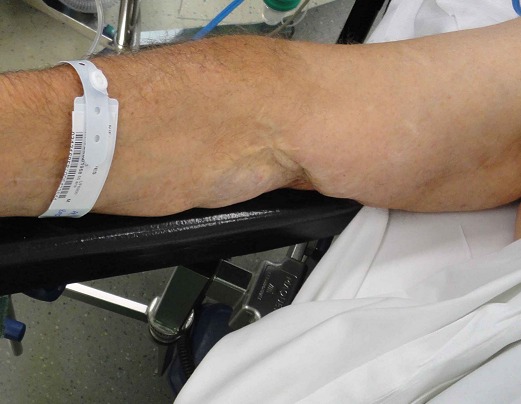
Deformed and amyotrophic right upper limb due to radial nerve palsy complicating humeral communitive fracture

**Figure 2:**
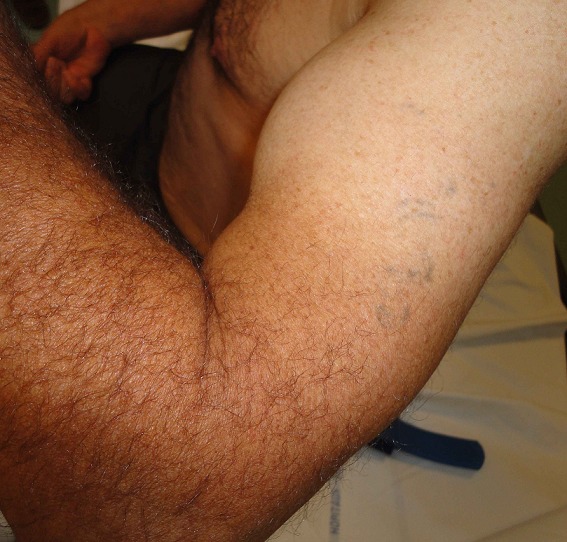
“Popeye's sign” pathognomonic of distal biceps tendon rupture

**Figure 3:**
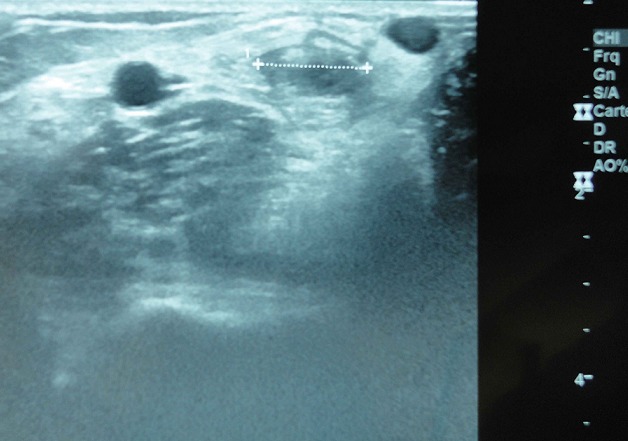
Ultrasonography of the elbow showing distal biceps tendon defect with hematoma around the humeral trochlea

**Figure 4:**
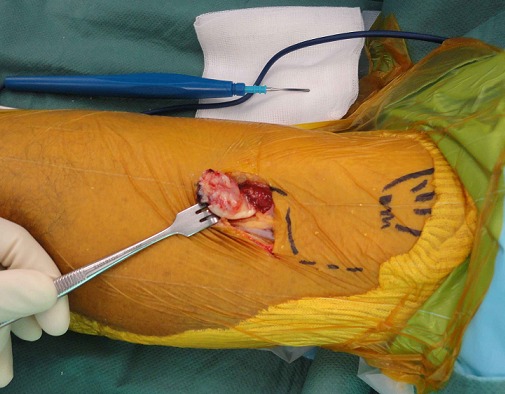
Single anterior approach of the elbow

**Figure 5:**
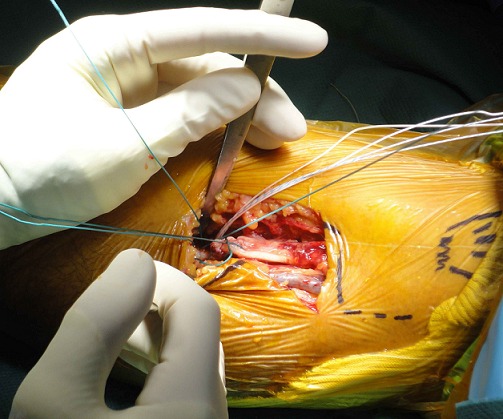
Trans-osseous anchoring of the distal biceps tendon in bicipital tuberosity

**Figure 6:**
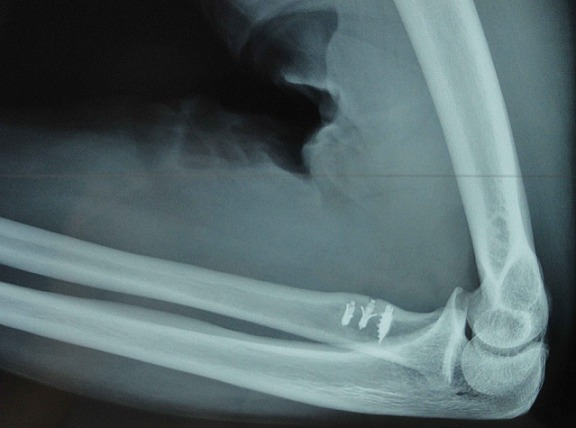
Lateral radiograph of the elbow showing trans-osseous anchors position

A 57 years old man, left-handed, admitted to department of orthopedics at Chambéry Hospital, France, for distal biceps rupture of the left arm after lifting a heavy object. We observed deformed and amyotrophic right upper limb due to radial nerve palsy complicating humeral communitive fracture (Figure 1). The clinical exam revealed “Popeye's sign” (Figure 2) and palpable mass in the mid-upper arm after flexion of the elbow. The hook test and squeeze test revealed complete rupture. Anteroposterior and lateral radiographs of the elbow didn't show bicipital tuberosity avulsion. Ultrasonography of the elbow showed intra-tendinous distal biceps defect with hematoma around the humeral trochlea (Figure 3). The patient was operated using single anterior approach (Figure 4) and three trans-osseous anchoring in correct tension (Figure 5 and Figure 6). A splint has been used to immobilize the left upper limb and protect the tendon repair for 6 weeks. Passive and progressive rehabilitation, ranged from 30° to 90° of flexion-extension, was encouraged during this period. Excellent clinical and radiological results were obtained after 4 months.

## Discussion

Acute distal biceps rupture represents 3% of biceps injuries [1]. The first description was reported by Acquavica in 1898. Adult and active persons in the fourth decade of life are more concerned and the dominant limb is often affected. Safran et al. report an incidence of 1.24 per 100,000 annually and suggest that smoking may increase the risk [2]. The mechanism of injury is an unexpected extension force applied to the flexed arm, such as lifting a heavy object. Clinically, patients report feeling a sudden painful tearing sensation in the antecubital region of the elbow; tenderness in the antecubital fossa, and a defect usually can be palpated there; supination test (supination against resistance) is helpful in making the diagnosis. Radiographs must be realized, it can show avulsion of a portion of the radial tuberosity. Magnetic resonance imaging (MRI) can be helpful to distinguish complete from partial ruptures. The treatment of acute distal biceps rupture is controversial; if therapeutic abstention provide poor results (pain, 30% loss of flexion strength and 40% limitation of supination strength [3]); surgical repair, particularly trans-osseous anchoring, guarantee good results in term of flexion and supination [4]. In fact, non-operative treatment can be recommended for elderly, sedentary patients who do not require strength in flexion and supination and for patients with medical problems. Surgical repair is the only option for regaining normal strength in the affected arm, typically in active and young adult. Two methods are commonly employed. The first, uses trans-osseous anchoring through two incisions; this approach was described by Boyd and Anderson to minimize anterior exposure and limit the risk to neurovascular structures [5], but the development of heterotopic ossification and radioulnar synostosis were frequent [6]. The second method, consists of a single anterior extensive approach; nowadays, the use of suture anchors makes it simpler and safer, and reduces the risks of both neurological injury and heterotopic ossification [7], many authors recommend fixation using two anchors because it's stronger than single trans-osseous repair [8]. In our case, using one-incision method with three trans-osseous anchors were our reference. Postoperative rehabilitation is fundamental to restore complete elbow function after 6 weeks of immobilization. Passive and progressive flexion-extension exercises are encouraged until full extension restore. Supination and pronation exercises are begun at 4 weeks. Active-assisted flexion and supination exercises are started at 8 weeks. For our patient, return to full activity was possible after 16 weeks.

## Conclusion

Surgical repair should be indicated in young active individuals in order to restore full strength in both flexion and supination, especially, when the dominant limb is affected. We encourage orthopedists to practice single anterior approach with trans-osseous anchoring for repairing acute distal biceps tendon rupture.

## References

[1] Gilcreest EL, Albi P (1939). Unusual lesions of muscles and tendons of the shoulder girdle and upper arm. Surg Gynec Obstet..

[2] Safran MR, Graham SM (2002). Distal biceps tendon ruptures: incidence, demographics, and the effect of smoking. Clin Orthop Relat Res.

[3] Morrey BF, Askew LJ, An KN, Dobyns JH (1985). Rupture of the distal tendon of the biceps brachii. A biomechanical study. J Bone Joint Surg..

[4] Woods DA, Hoy G, Shirmnin A (1999). A safe technique for distal biceps repair using a suture anchor and a limited anterior approach. Injury..

[5] Boyd HB, Anderson LD (1961). A method for reinsertion of the distal biceps brachii tendon. J Bone Joint Surg Am..

[6] Failla JM, Amadio PC, Morrey BF, Beckenbaugh RD (1990). Proximal radioulnar synostosis after repair of distal biceps brachii rupture by the two-incision technique. Clin Orthop Relat Res.

[7] D'Alessandro DF, Shields CL, Tibone Chandler JE (1993). Repair of distal biceps tendon ruptures in athletes. Am J Sports Med.

[8] Berlet GC, Johnson JA, Milne AD, Patterson SD, King GJ (1998). Distal biceps brachii tendon repair. An in vitro biomechanical studyof tendon reattachment. Am J Sports Med..

